# Assessment of Digital and Community-Based Outreach Interventions to Encourage COVID-19 Vaccination Uptake in an Underserved Community

**DOI:** 10.1001/jamanetworkopen.2022.17875

**Published:** 2022-06-22

**Authors:** Daniela Diaz, Sharon Chacko, Anne Sperling, Elaine Fleck, Irene Louh, Richard Trepp, Siqin Ye

**Affiliations:** 1 NewYork-Presbyterian Hospital, New York; 2 Department of Medicine, Columbia University Irving Medical Center, New York, New York; 3 Department of Emergency Medicine, Columbia University Irving Medical Center, New York, New York

## Abstract

This cohort study investigates changes in the proportions of racial and ethnic groups among COVID-19 vaccine recipients before and after digital and community-based outreach interventions.

## Introduction

Non-Hispanic Black (hereafter, Black) and Hispanic patients have higher risk for COVID-19 infection and hospitalization,^1,2^ but have lower rates of COVID-19 vaccination^3^ because of factors such as limited access to care, lack of outreach, technology and language barriers, and mistrust of health systems.^4^ To address this, we conducted 2 concurrent interventions at the NewYork-Presbyterian Hospital’s vaccination site at the Armory, located in a racially and ethnically diverse neighborhood in Northern Manhattan: (1) a digital redesign to restrict online self-scheduling for vaccination to local zip codes with underserved racial and ethnic minority patient populations^4^ and (2) direct outreach to educate and schedule patients through community-based organizations (CBO).^4,5^ Here we describe changes in race and ethnicity makeup of COVID-19 vaccine recipients before and after these interventions.

## Methods

The Armory vaccination site operated from January 14, 2021, to May 14, 2021. Online self-scheduling via the patient portal (Epic Systems) was initially enabled for all New York State residents. Beginning January 28, 2021, self-scheduling was restricted to residents from local zip codes with high racial and ethnic minority populations. In parallel, direct outreach with a focus on local Spanish-speaking and underserved communities was conducted through CBOs, such as community and senior centers, faith-based organizations and local primary care practices, beginning on February 3, 2021.^5^ Components of outreach included social media campaigns, vaccine ambassador programs, presentations, and enabling CBOs to directly schedule and reserve walk-in slots.

The Columbia institutional review board approved waiver of informed consent due to the study being minimal risk. We extracted scheduling data and analyzed demographic characteristics of all patients who received at least 1 COVID-19 vaccination at the Armory site, including self-reported race and ethnicity and zip code. The daily proportion of patients receiving a first-dose COVID-19 vaccination who were from a racial and ethnic minority population was tabulated. We followed the Strengthening the Reporting of Observational Studies in Epidemiology (STROBE) reporting guideline for cohort studies. χ^2^ tests were used to assess differences in race and ethnicity categories between patients who self-scheduled prior to zip code restrictions, those who self-scheduled after, and those scheduled through CBO outreach. Two-sided *P* < .05 was considered statistically significant. Statistical analysis was performed using Stata version 16 (StataCorp).

## Results

**Figure 1.**
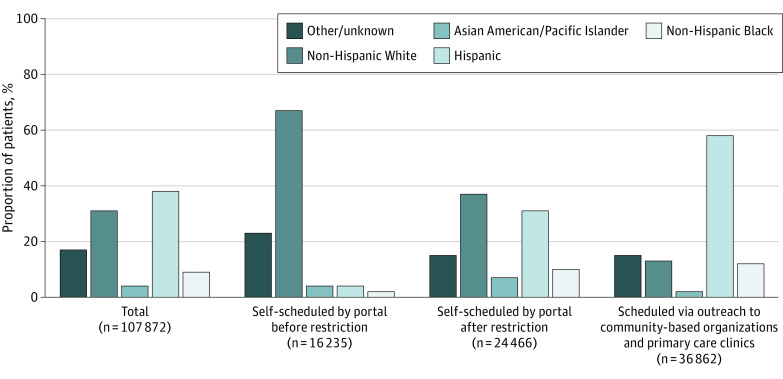
Race and Ethnicity of Patients Receiving First-Dose COVID-19 Vaccination The category of other or unknown included patients who self-reported a race or ethnicity that was not in one of the listed categories or if they selected “other” or “unknown” or declined to report race and ethnicity.

**Figure 2.**
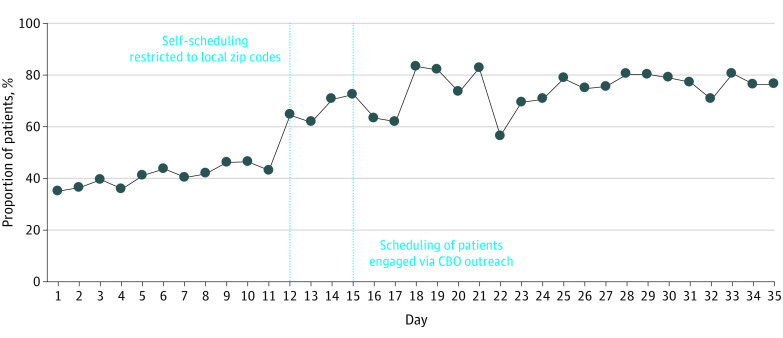
Daily Proportion of Patients Receiving First-Dose COVID-19 Vaccinations Who Were From Minoritized Racial and Ethnic Groups Vertical dashed lines represent when interventions began (day 12 for local zip codes self-scheduling restriction and day 15 for scheduling of patients engaged through outreach to community-based organizations [CBOs]).

A total of 107 872 patients received at least 1 dose of COVID-19 vaccine at the Armory mass vaccination site. Of these, 61 717 (57%) were female, 10 215 (9%) were Black, 40 879 (38%) were Hispanic, and the mean (SD) age was 57.8 (18.2) years. After the interventions were implemented, there was an immediate and sustained increase in the proportion of racial and ethnic minority patients receiving first-dose COVID-19 vaccination (Figure 1). Black patients represented 4291 of 36 862 patients (12%) scheduled through CBO outreach, 2508 of 24 466 patients (10%) self-scheduled after local zip codes restriction, and 290 of 16 235 patients (2%) self-scheduled prior to restriction. Similarly, Hispanic patients represented 21 484 of 36 862 patients (58%) scheduled through CBO outreach, 7521 of 24 466 patients (31%) self-scheduled after local zip codes restriction, and 712 of 16 235 patients (4%) of those self-scheduled prior to restriction (*P* < .001 for all pairwise group comparisons) (Figure 2).

## Discussion

In this analysis of data from a mass COVID-19 vaccination site, the proportion of Black and Hispanic patients receiving the COVID-19 vaccine was substantially higher with CBO outreach than when self-scheduled through the patient portal. Restriction to local zip codes for self-scheduling was also associated with a higher proportion of racial and ethnic minority patients receiving the COVID-19 vaccine. These findings suggest that direct outreach from trusted community resources can address challenges navigating self-scheduling technology^6^ and may mitigate distrust of COVID-19 vaccination in Hispanic and Black communities.

Limitations of our study include its nonrandomized, observational design, and the use of a convenience sample from a single site at the beginning of the COVID-19 vaccine rollout that may limit generalizability. Nonetheless, our results highlight the importance of direct, community-based engagement and appropriate digital workflow design for vaccination and public health campaigns and suggest that such efforts can potentially mitigate health disparities.
